# Assessing the Level of Prostate Cancer Care in a Low‐Resource Setting: Disease Pattern, Current Situation of Care, and the Need for Reform—A Retrospective Observational Study From East Oncology Centre, Sudan

**DOI:** 10.1155/aiu/3967008

**Published:** 2026-09-15

**Authors:** Mosab A. A. Alzubier, Eltahir Ahmed Eltahir, Hind Mohi Aldin Abd Allah, Mutasim Mursi Abubaker, Safia Ibrahim Omer Khalid, Abuzar Awadalla Ibrahim Elkhidir Abdol, Mushrga Alamin Ahmed Daood

**Affiliations:** ^1^ Department of Surgery, Faculty of Medicine, Prince Sattam Bin Abdulaziz University, Al Kharj, Saudi Arabia, psau.edu.sa; ^2^ Department of Surgery, Faculty of Medicine, Gadarif University, Gadarif, Sudan; ^3^ Department of Surgery, Faculty of Medicine, AL Fashir University, AL Fashir, Sudan; ^4^ Faculty of Medicine and Dentistry, Western Campus, Kampala International University (KIU), Bushenyi, Uganda; ^5^ Ministry of Health, East Oncology Centre, Gadarif, Gadarif State, Sudan, sante.gov.ma

**Keywords:** East oncology centre, prostate cancer, suboptimal care, Sudan

## Abstract

**Background and Aims:**

Prostate cancer (PC) is a common male malignancy, and it is often diagnosed at advanced stages, particularly in developing countries, largely due to underdeveloped healthcare systems. Resource‐limited care, such as bilateral orchidectomy for symptomatic illness, is an economically feasible therapeutic option that may enhance patient condition and survival in a low‐resource situation. This study aims to assess the status of PC care and treatment outcomes in eastern Sudan.

**Methods:**

A retrospective hospital‐based study was conducted at the East Oncology Centre (EOC), Gadarif State, eastern Sudan, including all patients diagnosed with PC between May 2016 and December 2023. Relevant demographic, clinical, pathological, treatment, and outcome data were retrieved from patients’ medical records.

**Results:**

A total of 231 patients with PC, with a mean age of 70.9 years (± SD 10), all of whom were Sudanese from different ethnic groups, were included in the study. Most patients (86.6%; *n* = 200) presented with lower urinary tract symptoms, and only 15.6% had a family history of PC. Most of the patients (62.34%, *n* = 144) had prostate‐specific antigen (PSA) levels ranging from 21 to 100 ng/mL; histopathology revealed adenocarcinoma of the prostate with a Gleason score of 8 or more in 60.6% (*n* = 140). Bone scan results were positive in 50.2% of cases, and 88.3% (*n* = 204) received hormonal treatment as the primary modality for cancer control. The mortality data indicate that 24.7% (*n* = 57) of patients had died.

**Conclusions:**

This study highlights the suboptimal care for PC in eastern Sudan, where most patients present with late‐stage disease, probably due to resource limitations. Addressing late‐stage PC requires opportunistic digital rectal examination screening, targeted PSA testing, and increased awareness. Improved outcomes rely on specialist training, standardised care protocols, and the expansion of radiation facilities.

## 1. Introduction

Prostate cancer (PC) is among the most common cancers affecting men worldwide [1]. Due to its slow progression, PC often remains asymptomatic until it advances, resulting in delayed diagnosis at the late stages. [2]. In developing countries, most patients present with advanced disease, largely due to underdeveloped healthcare systems [3]. This is attributed to challenges such as limited access to healthcare, lack of early detection services, and infrequent routine PC screening by physicians, often due to limited knowledge or constrained resources, which make prostate‐specific antigen (PSA) testing costly or unavailable [2, 4, 5]. Although there is ongoing debate regarding the effectiveness of PSA screening [6], evidence suggests that PSA screening can reduce the incidence of advanced disease and PC‐related mortality [1, 7].

In resource‐limited settings, treatment selection is often constrained by the availability of therapeutic modalities rather than by clinical preference alone [2]. Although it can be difficult to pinpoint the exact timing, androgen deprivation therapy (ADT) is a key component of PC treatment, with roughly 50% of patients receiving ADT at some stage [8, 9]. Resource‐limited care, such as bilateral orchidectomy for symptomatic illness, is an economically feasible therapeutic option that may enhance patient condition and survival in a low‐resource situation [2].

The aim of this study is to assess the current patterns and quality of PC diagnosis, management, and outcomes at the East Oncology Centre (EOC) in eastern Sudan and to identify gaps in PC care within a resource‐limited healthcare setting.

The EOC in Gadarif State, Eastern Sudan, was established in 2015 and began functioning in 2016. It is one of the 12 cancer centres set up across Sudan between 2008 and 2021 as part of a national effort to expand oncology services. The Gadarif Centre provides medical oncology services, which primarily include chemotherapy, hormonal therapy, and palliative care. More advanced treatments, like radiation therapy, are not yet available.

Patients requiring radiotherapy were routinely referred to other oncology centres where these services were available. Following completion of radiotherapy, many patients returned to the EOC to continue their follow‐up and ongoing oncological care.

To our knowledge, this is the first study to assess the status of PC diagnosis, treatment, and outcomes in eastern Sudan. The purpose of this study is to assess the state of PC care to facilitate the development and implementation of future policies that standardise practice to enhance patient care and treatment outcomes.

## 2. Methods

This retrospective, observational, descriptive study was conducted at EOC in Gadarif State, eastern Sudan, to evaluate the management and outcomes of patients with PC. Data were collected using a Google Form questionnaire specifically designed for this study. The questionnaire included demographic data and data on clinical presentation, diagnostic workup, treatment, and outcomes.

The data were extracted retrospectively from patient medical records between May 2016 and December 2023. All consecutive patients diagnosed with PC and managed at the EOC between May 2016 and December 2023 were eligible for inclusion in the study. Patients with incomplete records that precluded the extraction of essential demographic or clinical information were excluded from the relevant analyses. Histopathological confirmation of PC was available for most patients; however, a small number of patients were diagnosed based on highly suggestive clinical findings, markedly elevated PSA levels, and digital rectal examination (DRE) findings when biopsy was not feasible. Trained members of the research team performed data extraction. To ensure data quality, the principal investigators independently reviewed the extracted data for completeness and consistency and resolved any discrepancies by re‐examining the original medical records before statistical analysis.

Clinical progression was defined as documented radiological progression, worsening clinical symptoms attributable to disease progression, or physician‐documented progression requiring treatment modification.

Biochemical recurrence was defined based on available post‐treatment PSA follow‐up documentation; however, standardised biochemical recurrence criteria could not always be applied.

Mortality was defined as death documented in medical records or follow‐up records, irrespective of cause.

The collected data were analysed using Stata Statistical Software: Release 17 (StataCorp, 2021). Descriptive statistics were used to summarise the demographic and clinical variables.

The study was approved by the Medical Ethics Committee of the University of Gadarif, Faculty of Medicine, Sudan (reference: RC. Q4.5.5.24). Patient confidentiality was maintained throughout the study by anonymising all data before analysis.

## 3. Results

### 3.1. Demographic Data

A total of 231 patients diagnosed with PC were included in the study. The mean age of the patients was 70.9 years (± SD 10), with an age range of 44–100 years. More than half of the patients (54.1%) resided in rural areas. Among the cohort, 209 patients (90%) were married, 11 (4.8%) were widowed, 3 (1.3%) were single, and 8 (3.5%) were divorced. All participants were Sudanese, representing various ethnic groups.

### 3.2. Patterns of Presentation

**FIGURE 1:**
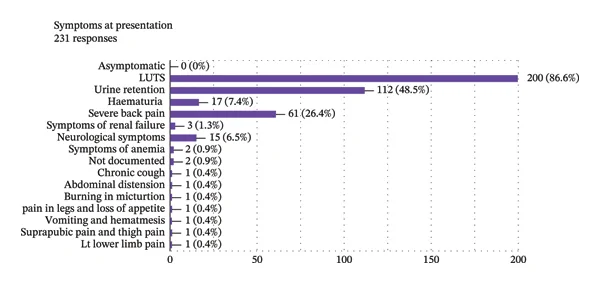
Clinical symptoms at presentation.

Most patients (86.6%, *n* = 200) presented with lower urinary tract symptoms (LUTS), with or without other associated symptoms, while the remaining patients primarily presented with other symptoms in addition to LUTS (Figure 1).

Nineteen percent (*n* = 44) of patients were diagnosed with benign prostate enlargement (BPE) and received treatment before being diagnosed with PC. 13% (*n* = 30) of patients were hypertensive, and 6.9% (*n* = 16) were diabetic. Only 15.6% of patients had a family history of PC. Smoking was reported in 33 patients (14.3%), and alcohol consumption in nine patients (3.9%). Six patients (2.6%) were both smokers and alcoholics, and 183 patients had habits not stated (Table 1).

**TABLE 1:** Patient characteristics.

	Freq.	Percentage
*Chronic Diseases*		
Hypertension	30	13%
Diabetes mellitus	16	6.9%

*Family History of Prostate Cancer*		
No	195	84.42%
Yes	36	15.58%

*Habits*		
Smoking	33	14.29%
Alcohol	9	3.90%
Smoking and alcohol	6	2.60%

*BPH Prior to PC diagnosis*		
No	129	55.84%
Yes	44	19.04%
No data documented	58	25.1%

*DRE Findings*		
Not documented or not done	192	86.1%
Nodular or hard prostate	37	16.02%
Normal or enlarged prostate with benign features	2	0.87%

**FIGURE 2:**
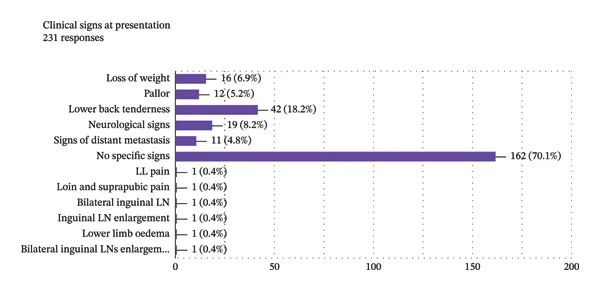
Clinical signs at presentation.

Figure 2 shows that in a substantial portion of the cohort, no specific clinical signs were recorded at the time of presentation (70%, *n* = 162); however, lower back tenderness was the most frequently reported sign, occurring in 42 cases (18.2%), followed by neurological signs (8.2%, *n* = 19) and loss of weight (6.9%, *n* = 16). Regarding DRE, 37 patients (16%) had a nodular or hard prostate, and only 0.86% had a normal‐feeling prostate. DRE was not documented or not done in 192 patients (83.1%) (Table 1).

### 3.3. Investigations

PSA level was recorded in 215 patients; most patients (62.3%, *n* = 144) had PSA levels ranging from 21 to 100 ng/mL. A total of 45 participants (19.5%) presented with PSA levels greater than 100 ng/mL, and only 16 patients (6.9%) had PSA levels less than 10 ng/mL (Table 2).

**TABLE 2:** Different investigations, PSA, USS, histopathology, and bone scan.

	Freq.	Percentage
*PSA level (ng/mL)*		
21 to 100	144	62.34%
More than 100	45	19.48%
Less than 10	16	6.93%
10 to 20	10	4.33%
Not available or not documented	16	6.93%

*USS Finding*		
Prostate enlargement	195	84.42%
Ureteric obstructions	32	13.85%
Pelvic LN	23	9.96%
Not documented	24	10.39%
Normal USS findings	7	3.03%
UB mass	2	0.87%
RT kidney cyst	1	0.43%
Liver metastatic lesion	1	0.43%
Vesical stone	1	0.43%

*Gleason Score*		
9 or 10	91	39.39%
8	49	21.21%
4 + 3	27	11.69%
3 + 4	17	7.36%
Less than 7	16	6.93%
Not available or not documented	31	13.42%

*Bone Scan Result*		
Positive	116	50.22
Not done	77	33.33
Negative	38	16.45

Table 2 shows the findings of the ultrasonographic (USS) study, which was recorded in 207 cases (89.6%). Prostate enlargement was the most common finding, observed in 195 cases (84.4% of the total), with a mean volume of 88.1 cc. Other findings in USS include ureteric obstruction in 32 cases (13.9%) and enlarged pelvic lymph nodes in 23 cases (10%).

Histopathology was done in nearly all cases (97%; *n* = 224), and only 7 (3%) cases were diagnosed based on high PSA and DRE; however, the Gleason score was recorded in 200 cases (86.5%) and revealed that only 6.9% (*n* = 16) scored less than 7, 44 (19%) patients scored 7, and the majority (60.6%, *n* = 140) scored 8 or more. Bone scan results were positive in 50.2% of cases and negative in 16.4%, while in 33.3%, it was not done (Table 2).

### 3.4. Treatment Modalities

The median duration between diagnosis and treatment initiation was 10 days (Table 3). Table 4 shows that most patients, 88.3% (*n* = 204), received hormonal treatment as the primary modality for cancer control. A smaller subset received a combination of hormonal treatment with radiotherapy (4.8%, *n* = 11), chemotherapy (4.3%, *n* = 10), or both radiotherapy and chemotherapy (1.3%). Only 2 patients (0.87%) underwent radical prostatectomy in conjunction with hormonal treatment.

**TABLE 3:** Treatment outcomes and time durations.

Treatment outcomes						
Variable	Yes		No		Not documented	
Clinical Progression	78	33.8%	82	35.5%	71	30.7%
Biochemical Recurrence	59	25.5%	80	34.6%	92	39.8%
Mortality	57	24.7%	56	24.2%	118	51.1%

*Median Time Duration Between (in days)*						
Diagnosis to Treatment Initiation					10 days	
Treatment to Clinical Progression					406 days (13.5 months)	
Treatment for Biochemical Recurrence					538 days (18 months)	
Follow‐up Duration					390 days (13 months)	

**TABLE 4:** Treatment modalities.

Treatment Applied for Cancer Control	Freq.	Percentage
Hormonal treatment	204	88.3
Hormonal treatment, Radiotherapy	11	4.76
Hormonal treatment, Chemotherapy	10	4.33
Hormonal treatment, Chemotherapy, Radiotherapy	3	1.30
Radical prostatectomy, Hormonal treatment	2	0.87
Chemotherapy	1	0.43

The time to PSA nadir, an important marker of treatment response, varied significantly among patients. While 41.9% of patients had no documented data, 21.6% reached a nadir within 4–6 months. A significant proportion (21.1%) of patients experienced persistently high PSA levels, and 15.4% reached nadir after more than 6 months.

**FIGURE 3:**
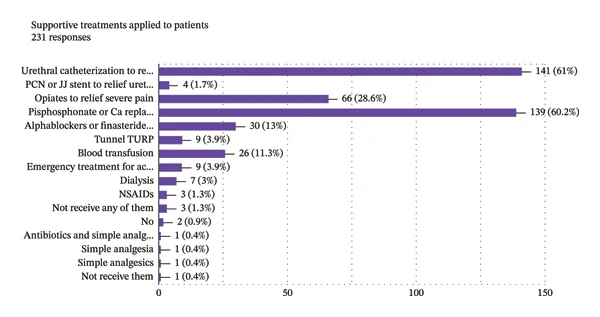
Palliative treatments applied to patients.

Figure 3 summarises the supportive and palliative interventions provided to patients during treatment. Urinary diversion procedures, pain management, blood transfusion, and palliative transurethral resection of the prostate were among the most required supportive measures, reflecting the substantial supportive care needs of this patient population.

## 4. Outcomes

Among the cohort, 35.5% (*n* = 82) of patients experienced no clinical progression, while 33.8% (*n* = 78) had disease progression. However, 30.7% (*n* = 71) had no data available on progression status, suggesting potential gaps in follow‐up or documentation. For biochemical recurrence, 39.8% (*n* = 92) of patients lacked data. Among those with available data, 34.6% (*n* = 80) had no recurrence, and 25.5% (*n* = 59) experienced biochemical recurrence, indicating disease relapse in a significant proportion of patients. Mortality data were available for 113 (48.9%) patients and were missing for 118 (51.1%). Of the total cohort, 57 patients (24.7%) had documented deaths, and 56 (24.2%) were documented as alive at the last follow‐up. The substantial proportion of missing recurrence and mortality data limits interpretation of outcomes. The median follow‐up duration was 13 months (Table 3).

## 5. Discussion

PC is the fifth‐leading cause of cancer‐related mortality in men globally as well as the second among all male malignancies [10]. In Sudan, PC is the most prevalent malignancy in men, equal in incidence across all tribes, and 85.4% of patients had stage 3 or stage 4 disease at presentation [11]. Through cancer screening, substantial progress has been made in early detection [12]. However, men of lower socioeconomic class continue to have a significant incidence of high‐stage and high‐grade PC illness [13], even though PC stage and grade have been shown to shift towards the identification of lower risk diseases. This is largely due to a lack of knowledge and early detection services [1, 2]. Achieving prompt diagnosis and guaranteeing access to fair and affordable treatment are challenging issues [14], especially in low‐resource settings.

Nearly every patient in this study had clinical findings suggestive of advanced illness. This is possibly attributed to the lack of a programme to screen such individuals and to the unavailability and unclear policy on utilising and interpreting diagnostic instruments, such as PSA, which reflects the condition of care and medical services offered to those at risk of having PC. Since 19% of patients in this study had BPE treatment before receiving a PC diagnosis, the notion that BPE is the only cause of LUTS may partially explain the late presentation of PC patients. Furthermore, we discovered that in 86% of instances, DRE was neither performed nor documented in patient records, suggesting that not utilising DRE as a routine test in the evaluation of patients with LUTS may contribute to delays in identifying significant PC. This also results in inadequate patient staging.

Most patients (86.6%) had LUTS at presentation, and according to current guidelines, the PSA test must be included in the LUTS workup [15]. Significantly elevated PSA values, which define participants as high risk, were found in the majority of participants (62% had PSA levels between 21 and 100 ng/mL, and 19.5% had PSA levels above 100 ng/mL).

This is associated with the high disease grade identified by biopsy (more than 60% have a Gleason score of 8 or more) and, according to the bone scan results (although not performed in all patients), 50% were metastatic at presentation.

A thorough risk stratification was not possible, as patient records lacked information on accurate disease staging, even though most patients demonstrated features suggestive of advanced disease. To properly apply guidelines, additional clinical training and technological equipment are required to increase the completeness of PC staging [2]. By reducing the need for ADT and radiation, lower stage disease improves treatment outcomes and quality of life [16]. Moreover, it improves survival in developed countries because early diagnostic techniques and effective treatment options are available [17]. Compared with localised illness, treating metastatic PC may be more expensive [4], and despite improvements in treatment methods, the prognosis is worse for patients with distant metastatic disease at presentation [13].

For various PC stages, several treatments are available; however, opinions on the optimal sequence or combination remain divided [18, 19]. The decision‐making process must weigh the advantages, life expectancy, comorbidities, possible adverse effects of therapy, and other logistic factors [19]. The availability of treatment options and the professionals’ clinical experience should also be considered in our case.

The documented data of high PSA levels, high disease grade in biopsy results, and a relatively high rate of positive bone scan results would strongly prove that most patients demonstrated features suggestive of advanced disease, at least locally advanced disease, if not metastatic, at the time of presentation, making curative treatment options difficult or inapplicable.

ADT alone is no longer regarded as adequate treatment for metastatic hormone‐sensitive prostate cancer (mHSPC), even though it is the mainstay in the management of patients with this disease [20]. This is because several pieces of evidence indicate that additional treatment significantly improves overall survival and progression‐free survival in mHSPC [21]. ADT with docetaxel or second‐generation hormonal treatment is the current standard of care for treating mHSPC [22]. On the other hand, little is currently known about the optimal combination and sequence of agents [21].

Most patients (88%) in this study received ADT as the primary treatment modality due to the limited availability of novel systemic therapies, the absence of radiotherapy services at the EOC, and low patient compliance. Patients requiring radiotherapy were routinely referred to other oncology centres where these services were available. Following completion of radiotherapy, many patients returned to the EOC to continue their follow‐up and ongoing oncological care. Despite these referral arrangements, access to radiotherapy remained challenging due to travel requirements, financial constraints, and treatment delays, underscoring the need to expand radiotherapy services in eastern Sudan.

The considerable need for supportive and palliative interventions further suggests that many patients presented with symptomatic advanced disease requiring multidisciplinary supportive care in addition to cancer‐directed treatment. This finding underscores the importance of integrating comprehensive palliative care services into PC management in resource‐limited settings.

Despite the relatively short median time (10 days) between diagnosis and treatment initiation, which suggests that EOC policymakers have worked hard, the high percentage of missing data on disease recurrence and mortality rates, often due to loss to follow‐up, also reflects low patient compliance. The median interval between ADT and relapse, based on the available information, was 13.5 months for clinical progression and 18 months for biochemical recurrence. These durations are comparable to those reported in the literature, which ranged from 12 to 24 months [23, 24].

Among patients with documented mortality status, approximately half had died during follow‐up; however, because mortality information was unavailable for more than half of the cohort, these findings should be interpreted cautiously and should not be considered an estimate of overall mortality in this population. This finding is similar to those of research conducted by Seraphin et al., with roughly the same number of resources carried out in Sub‐Saharan Africa [25].

The interpretation of treatment outcomes should be undertaken with caution because a substantial proportion of patients had incomplete follow‐up data, particularly for clinical progression, biochemical recurrence, and mortality. The extent of missing outcome information may have introduced follow‐up bias and limited the precision of the reported outcome estimates. Consequently, the outcome data presented in this study should be considered descriptive rather than definitive and interpreted within the context of these limitations.

According to this study, the care situation for PC patients in eastern Sudan is beset by several challenges, including a lack of resources and knowledge regarding the significance of early PC detection, the unavailability of cutting‐edge diagnostic, staging, and therapeutic modalities like MRIs, bone scans, radiation therapy, and innovative medications, as well as a lack of experience performing radical surgical procedures. Taken as a whole, these variables may partially explain the prevalence of late presentations and suboptimal treatment in many cases. Strong initiatives are required to raise public and medical professional awareness of PC. Furthermore, it is strongly advised to standardise practices for diagnosing, staging, treating, and monitoring PC patients according to the facilities available. Accurate data recording is essential for both assessing the current situation and implementing plans.

Although Sudan has established a national cancer registry framework, nationwide cancer registration remains incomplete, and coverage varies across regions. The EOC contributes institutional data to the national oncology system; however, the present study represents only patients diagnosed and managed at this referral oncology centre. Consequently, the study cohort may not capture all PC cases occurring in eastern Sudan or elsewhere in the country, particularly patients who were not referred for specialist oncology care. These factors should be considered when interpreting the findings, and the results should primarily be viewed as reflecting the pattern of PC care at a major regional referral centre rather than the overall national experience.

### 5.1. Limitations

Due to its retrospective design, which relies on the completeness and accuracy of preexisting medical data, this study on PC in EOC in eastern Sudan has several drawbacks. First, inadequate recording in many patient files was a significant issue, potentially resulting in the omission of important information, such as thorough clinical data (including the exact dates of symptom onset), disease staging, and follow‐up and outcome information. Second, because of the small number of patients treated with alternative modalities and the substantial proportion of missing follow‐up data, a meaningful comparative analysis of outcomes across treatment modalities would have been statistically unreliable and potentially misleading.

Third, detailed information regarding patients’ activities of daily living, quality‐of‐life measures, symptom burden and the extent to which palliative needs were met was not consistently documented in the medical records. Furthermore, the missing follow‐up data may have also introduced follow‐up bias and limited the reliability of the outcome analyses; therefore, these findings should be interpreted with caution. Finally, the study was limited to descriptive statistical analyses, which did not permit evaluation of associations between clinical variables or identification of predictors of treatment outcomes.

To provide more thorough insights into PC in this area, these limitations underscore the need for further prospective research and improved record‐keeping mechanisms.

## 6. Conclusions

The standard of care for PCs in eastern Sudan is shown in this study. The vast majority of patients presented with features suggestive of advanced disease, and their workup and treatment were comparatively constrained by available resources. To reduce the high proportion of late‐stage presentations, care providers should opportunistically screen for DRE, raise awareness of the condition and implement targeted PSA screening for higher risk individuals. To improve the outcomes of PC treatment in eastern Sudan, more radiation facilities and, eventually, highly qualified urological surgeons and radio‐oncologists are needed to provide curative treatment options. Depending on the available facilities, a consistent plan for treatment, follow‐up procedures, data recording and efficient patient communication should be ensured.

**Nomenclature**
PC: Prostate cancer
ADT: Androgen deprivation therapy
EOC: East Oncology Centre
LUTS: Lower urinary tract symptoms
PSA: Prostate‐specific antigen
DRE: Digital rectal examination
BPE: Benign prostate enlargement
mHSPC: Metastatic hormone‐sensitive prostate cancer

## Author Contributions

Mosab A. A. Alzubier, Eltahir Ahmed Eltahir, Hind Mohi Aldin Abd Allah and Mutasim Mursi Abubaker participated in the study’s conception and design. Hind Mohi Aldin Abd Allah, Abuzar Awadalla Ibrahim Elkhidir Abdol, Safia Ibrahim Omer Khalid and Mushrga Alamin Ahmed Daood collected the data, performed a literature review, partially interpreted the data and contributed to manuscript drafting. Mosab A. A. Alzubier and Eltahir Ahmed Eltahir drafted the manuscript. Mosab A. A. Alzubier conducted a critical review of the results’ interpretation, revised the manuscript to strengthen intellectual content and participated in the final writing.

## Funding

No specific grant was received from any public, commercial or nonprofit funding agency for this research.

## Disclosure

The authors affirm that this manuscript is an honest, accurate and transparent account of the study being reported; that no important aspects of the study have been omitted; and that any discrepancies from the study as planned (and, if relevant, registered) have been explained. All authors read the final manuscript and approved it.

## Ethics Statement

The study was approved by the Medical Ethics Committee of the University of Gadarif, Faculty of Medicine, Sudan (reference: RC. Q4.5.5.24).

## Conflicts of Interest

The authors declare no conflicts of interest.

## Data Availability

All datasets collected and analysed in this survey are available from the corresponding author upon any reasonable request.
